# B/T Mixed Phenotype Acute Leukaemia Harbouring *NUP98::BPTF* Fusion

**DOI:** 10.1002/jha2.70166

**Published:** 2025-10-14

**Authors:** Van Tuong Nguyen, Lina Han, Jing Xu, Miguel Cantu, Franklin Fuda, Samuel John, Tamra Slone, Weina Chen

**Affiliations:** ^1^ Department of Pathology University of Texas Southwestern Medical Center Dallas Texas USA; ^2^ Department of Paediatrics University of Texas Southwestern Medical Center Dallas Texas USA

**Keywords:** AML, B/T MPAL, cooperative gene alterations, mixed phenotype acute leukemia (MPAL), *NUP98* fusion, *NUP98::BPTF*, T‐ALL

## Abstract

**Background:**

*NUP98*::*BPTF* rearranged (r) leukaemia is rare with only five reported cases, including acute myeloid leukemia and T‐lymphoblastic leukemia (T‐ALL).

**Results:**

Herein, we report a case of *NUP98::BPTF*‐r mixed phenotype acute leukemia (MPAL), B/T with T‐lineage‐predominance in a 15‐year‐old female. Cytogenetic and molecular studies revealed a t(11;17)(p15;q23)/*NUP98::BPTF* fusion and cooperative gene alterations characteristic of T‐ALL (*NOTCH1*, *FBXW7*, and *PHF6*). Our patient had a primary induction failure with T‐ALL‐directed chemotherapy and achieved complete remission following consolidation.

**Conclusion:**

This is the first case study characterizing the clinicopathological and genomic features of B/T MPAL harboring *NUP98::BPTF* fusion and providing insights into molecular pathogenesis.

**Trial Registration:**

The authors have confirmed clinical trial registration is not needed for this submission.

**Abbreviations**
ALL: Acute lymphoblastic leukemia
AMKL: Acute megakaryoblastic leukemia
AML: Acute myeloid leukemia
AMML: Acute myelomonocytic leukemia
B‐ALL: B‐lymphoblastic leukemia
BM: Bone marrow
COG: Children's Oncology Group
CR: Complete remission
ETP‐T‐ALL: Early T‐cell precursor T‐lymphoblastic leukemia
HSCT: Hematopoietic stem cell transplant
ICC: The International Consensus Classification
KMT2A: Lysine Methyltransferase 2A (also known as MLL)
MPAL: Mixed phenotype acute leukemia
MRD: Measurable residual disease
NUP98: Nucleoporin 98
PB: Peripheral blood
PHD: Plant Homeodomain
SH2B3: SH2B Adaptor Protein 3
T‐ALL: T‐lymphoblastic leukemia
WHO‐HEM5: The fifth edition of the World Health Organization Classification of Tumours of the Hematopoietic and Lymphoid Tissues
WT1: Wilms Tumor 1

## 1 Introduction

Mixed phenotype acute leukemia (MPAL) is an aggressive leukemia in which leukemia blasts exhibit immunophenotypic evidence of differentiation along more than one hematopoietic cell lineage [1, 2]. Within this category, B/T MPAL is uncommon, accounting for only ∼5% of MPAL cases, and characterized by blasts expressing both B‐ and T‐lineage defining markers. Diagnosis and clinical management of MPAL are often challenging due to its rarity, immunophenotypic and genomic complexity, and lack of biologically informed treatment guidelines [3].

The *NUP98* gene (chromosome 11p15) encodes a nucleoporin protein that plays a role in molecular transport across the nuclear envelope. The NUP98 fusions involve the N‐terminus of the NUP98 protein and the C‐terminus of a partner (including *HOX* genes and non‐*HOX* genes) and result in impairing differentiation and enhancing self‐renewal of hematopoietic stem cells [4].

The *NUP98* fusions are present in ∼5% of childhood acute leukemia, most frequently as *NUP98::NSD1* in acute myeloid leukemia (AML), *NUP98::KDM5A* in acute megakaryoblastic leukemia (AMKL), and less commonly *NUP98::RAP1GDS1* and *NUP98::KDM5A* in T‐lymphoblastic leukemia (T‐ALL) [4, 5]. The NUP98 fusions have been recently reported in rare cases of MPAL, including *NUP98::NSD1* in B/Myeloid (M) MPAL, *NUP98*:*:MLLT1* in B/T MPAL, and *NUP98*:*IQCG* in T/M MPAL [6, 7, 8]. These leukemias are considered a high‐risk subtype of leukemia with poor prognosis, including induction failure [4].

Herein, we present the first case of B/T MPAL with T‐lineage predominance and harboring *NUP98::BPTF* fusion in a pediatric patient. This fusion is extremely rare with only five reported cases in the literature [5, 9, 10, 11, 12], spanning diverse phenotypes—AML [including AMKL and AMML (acute myelomonocytic leukemia)] and T‐ALL. This case study characterizes the clinicopathological features and cooperative gene alterations of B/T MPAL harboring *NUP98::BPTF* fusion and provides insights into leukemogenesis.

## 2 Result (Case Description)

**FIGURE 1:**
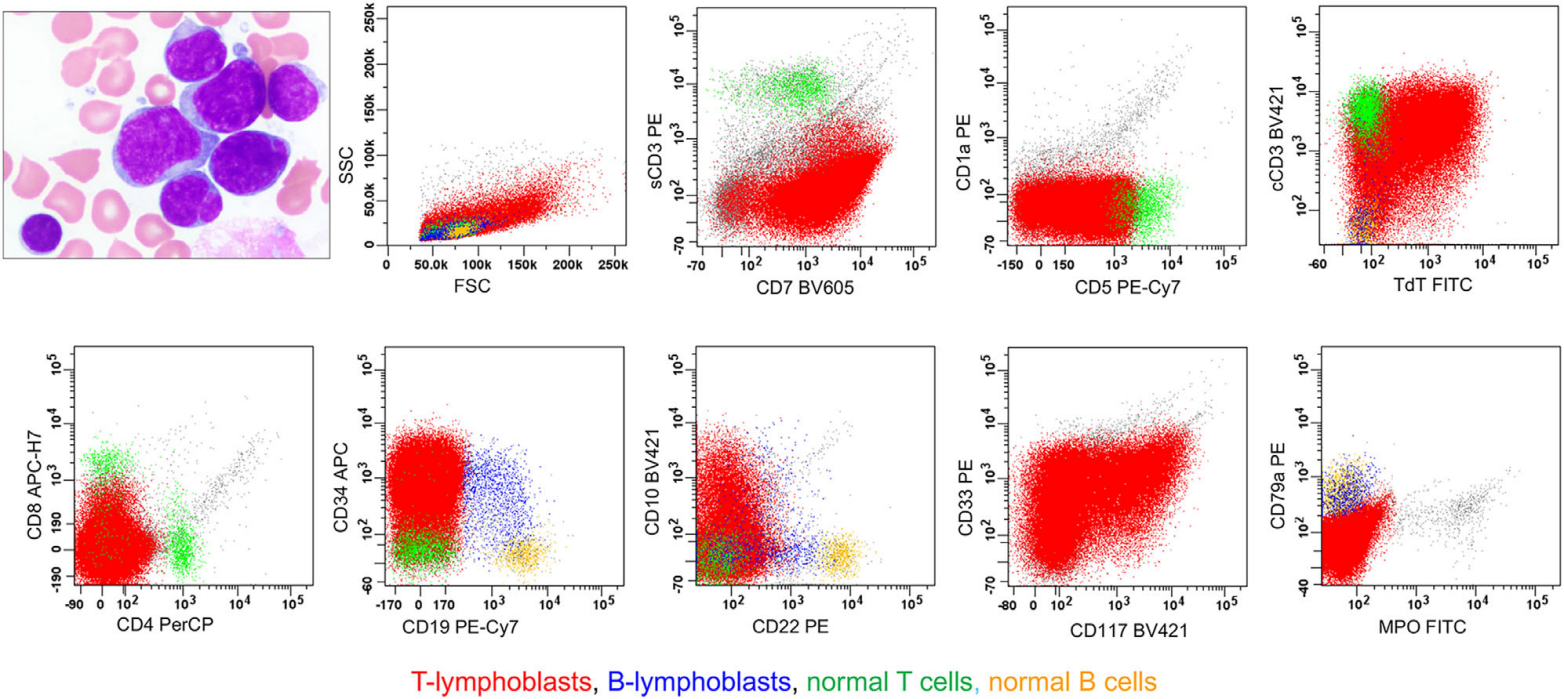
B/T MPAL (bilineal, consisting of T‐lymphoblasts and B‐lymphoblasts with T‐lymphoblast predominance). The upper left image demonstrates the morphologic features of lymphoblasts in BM aspirate: medium‐sized to large lymphoblasts with moderately condensed chromatin, irregular nuclei, small or inconspicuous nucleoli, and scant cytoplasm. By flow cytometric immunophenotyping (10‐color BD Canto and a panel of lymphoid/myelomonocytic markers [13]), there are two neoplastic populations of lymphoblasts, T‐lymphoblasts and B‐lymphoblasts. T‐lymphoblasts (in red) are predominant (92% of the total events), medium to large in size [with increased forward scatter (FSC)], and express cytoplasmic CD3 (cCD3), CD5 (partial), CD10(partial), CD7, CD33, CD34, CD117, and TdT, but lack expression of CD1a, surface CD3 (sCD3), CD4, CD8, CD19, or myeloperoxidase (MPO). This immunophenotype is consistent with ETP‐ALL (early T‐progenitor T‐ALL). B‐lymphoblasts (in blue) represent a minor population (0.8% of the total events), are medium‐sized (with low FSC), and express CD10 (a small subset), CD19, CD22 (partial), CD79a, CD34, and TdT (partial), but lack expression of sCD3, cCD3, or MPO.

A 15‐year‐old female with a history of scoliosis presented with two weeks of intermittent fever and worsening fatigue. Physical examination was unremarkable except for signs of anemia; no significant lymphadenopathy or organomegaly was noted. Laboratory evaluation revealed pancytopenia with a white blood cell count of 2.3 × 10^3^/µL with 45% blasts, hemoglobin of 3.0 g/dL, and platelet count of 56 × 10^3^/µL. Bone marrow (BM) examination revealed a hypercellular marrow with nearly complete replacement by medium‐sized to large blasts with moderately condensed chromatin, irregular nuclei, small or inconspicuous nucleoli, and scant cytoplasm (Figure 1). Flow cytometric analysis (BD‐Cantor, 10‐color panel [13]) identified two CD34(+)/TdT(+) neoplastic populations of lymphoblasts with a predominant population of T‐lymphoblasts (92% of the total events) that demonstrated characteristics of early T‐cell progenitor T‐ALL (ETP‐ALL), including expression of cytoplasmic CD3 (strong), CD117, and CD5 (partial) but absent surface CD3 and CD1a, and a separate minor population of B‐lymphoblasts (0.8% of the total events) (Figure 1). The immunophenotypic profile met the fifth edition of the World Health Organization Classification of Tumors of the Hematopoietic and Lymphoid Tissues (WHO‐HEM5) diagnostic criteria for MPAL, B/T subtype with a T‐lineage predominance, which permits the diagnosis of MPAL regardless of the percentage of the individual neoplastic blast population [1].

Conventional karyotyping revealed a t(11;17)(p15;q23) that resulted in the *NUP98::BPTF* fusion, confirmed by next‐generation sequencing (NGS by DNAseq and RNAseq on a BM sample), and additional abnormalities (second clone of 47, idem, +4). NGS also identified other gene alterations in *NOTCH1* (p.Leu1585Gln, VAF 27.61%)*, NOTCH1* (p.Leu1678Pro, VAF 16.76%)*, FBXW7* (p.Arg689Trp, VAF 22.88%)*, PHF6* (p.Arg274Gln, VAF 48.21%), and *SH2B3* (p.Pro432fs, VAF 27.80%) (Table 1). No rearrangements of *KMT2A, CRLF2*, or *BCR::ABL1* were present.

Given the T‐lineage predominance with only a minor population of B lymphoblasts, the patient was treated with T‐ALL‐directed induction chemotherapy (COG AALL1231) but showed refractory disease on day 29 BM with 60% T/B lymphoblasts with T‐lineage predominance. She was proceeded with consolidation chemotherapy per COG AALL0434 with nelarabine that led to measurable residual disease (MRD, 0.01% sensitivity) negative remission (4.3 months since diagnosis) with a plan to proceed with additional chemotherapy.

**TABLE 1:** Clinicopathologic and Genomic Features of Six Patients with *NUP98::BPTF* fusion.

Case (References)	Age/Sex	Cytogenetics	Other gene alterations	Chemotherapy	HSCT	A/D (follow‐up months)
AMML [10]	10 month/M	46, XY, t(11;17) (p15;q23)[20]	NA	Anthracycline/ cytarabine/ etoposide	Yes	A/(51)
AMKL [9]	8 month/M	49, XY, +6, +7, t(11;17)(p15;q23), +19	NA	Induction: low‐dose cytarabine, daunorubicin, etoposide, followed by Intensification and TVTC15^a^	NA	A/(NA)
AML [11]	20 year/F	46, XX, del(5)(q31), t(11;12)(p13;p13) [4] / 45, idem, ‐13 [5] / 46, XX [1]	*WT1, ETV6*	Induction 3+7^b^	NA	NA
T‐ALL [12]	19 year/F	86∼89, XXXX, +X, add(1)(p36), ‐4, ‐ 5, 6, ?add(6)(q21), ‐7, ‐8, ‐9, ‐10, ‐11, der(11)add(11)(p11) add(11)(q23), ‐13, ‐14, ‐15, ‐16, ‐17, ‐18, ‐18, ‐20, ‐21, +11, ∼15mar, inc [cp5] / 46, XX [14]	*JAK3*	ALL202‐U^c^	Yes	A (NA, CR)
AML [5]	0‐3/M	47, XY, +6, t(11;17)(p15;q23)[2]/48, idem, +6[10]/49, idem, +6, +21[2]/46, XY[6]	RNAseq: cluster in AMKL	NA	NA	NA
MPAL, T/B‐T (our patient)^d^	15 year/F	46, XX, t(11;17)(p15;q23)[6]/47, idem, +4[12]/46, XX[2]	DNAseq: *NOTCH1*, *FBXW7*, *PHF6*, and *SH2B3*	InductionAALL1231^e^; Consolidation AALL0434^f^	No	A/(4.3, CR^g^)

Abbreviations: +, positive; A/D, Alive/Died; AMKL, acute megakaryoblastic leukemia; AML, acute myeloid leukemia; T‐ALL, T‐lymphoblastic leukemia; AMML, acute myelomonocytic leukemia; CR, complete remission; F, female; HSCT, hematopoietic stem cell transplant, MRD, measurable residual disease; M, male; mo, months; MPAL, mixed phenotype acute leukemia; NA, not available; yr, years.

^a^Intensification: mitoxantrone and high‐dose cytarabine; third cycle: etoposide and high‐dose cytarabine; rescue treatment 5‐aza‐FLAG‐IDA (5‐azacitidine, fludarabine, high‐dose cytarabine, idarubicin, G‐CSF); TVTC15 Topotecan, Vinorelbine, Thiotepa and Clofarabine.

^b^Mentioned in Supplementary Table 1 of the Referenced Study, but no specific treatment details provided.

^c^Japan Adult Leukemia Study Group (JALSG)—ALL202‐U protocol [14].

^d^MPAL in this study, with (1) a predominant population of T‐lymphoblasts (∼99% of total blasts) and (2) a minuscule population of B‐ALL (∼1% of total blasts).

^e^ Vincristine, Daunorubicin, Pegaspargase, Methotrexate, and Dexamethasone.

^f^Cytarabine, Nelarabine, Cyclophosphamide, and 6‐Mercaptopurine.

^g^MRD (measurable disease detection) negative (0.01% sensitivity).

## 3 Discussion

This case study reports the first case of B/T MPAL harboring the *NUP98::BPTF* fusion and cooperative gene alterations, thus expanding the existing phenotypic spectrum of *NUP98::BPTF* rearranged leukemia (AML and T‐ALL) to include B/T MPAL. All six patients (5 patients reported previously [5, 9, 10, 11, 12] and our patient) were young with a median age of 8.3 years and male to female ratio of 1 and presented with various leukemia phenotypes, AML (including AMML and AMKL) in most patients (4/6), T‐ALL and B/T MPAL each in one patient (1/6). All patients had abnormal cytogenetic abnormalities, including most patients (4/6) with complex karyotype. Furthermore, *NUP98::BPTF* fusion was cytogenetically evident in most patients (4/6) by the presence of t(11;17)(p15;q23). Of four patients with clinical follow‐up, all patients (including our B/T‐T MPAL) exhibited an aggressive clinical course initially, including primary refractory disease or early relapse, consistent with the known association of *NUP98*‐rearranged leukemia with chemoresistance [4]. Three patients were proceeded with allogeneic hematopoietic stem cell transplant (allo‐HSCT) that led to a durable remission; our patient has a plan to receive HSCT. These four patients were alive at the last follow‐up.

The *NUP98::BPTF* fusion encodes a chimeric protein combining the FG‐repeat domains of *NUP98* with the PHD finger and bromodomain of *BPTF*. Alternative splicing yields variants with one or two PHD domains, and its expression is associated with activation of *HOXA9* and *HOXA10*, as well as upregulation of the proto‐oncogene *PIM1* [9, 11]. Structurally, it is similar to *NUP98::KDM5A*, as both fusions retain the FG‐repeat motifs of *NUP98* and the C‐terminal chromatin recognition modules of their partner proteins.

While *NUP98::BPTF* displays significant oncogenic potential by altering transcription and chromatin regulation, current evidence suggests that *NUP98* fusions are rarely sufficient on their own to induce leukemia [4]. Experimental models and clinical observations indicate that additional gene alterations, including *FLT3, NRAS, KRAS, KIT*, or *WT1* mutations, are typically required to provide the necessary proliferative or survival advantage [4]. This notion aligns with clinical observations across *NUP98*‐fusion leukemia, which almost invariably harbor cooperative gene alterations. Indeed, our case of *NUP98::BPTF* fusion positive B/T MPAL had cooperative gene mutations characteristic of T‐ALL (*NOTCH1*, *FBXW7*, and *PHF6*). Similarly, a case of *NUP98::BPTF* fusion positive AML had gene expression pattern clustered in AMKL by RNAseq [5]. These findings are similar to *NUP98::KDM5A* fusion positive leukemia, such as concurrent *NOTCH1* mutation in T‐ALL and *RB1* loss in AMKL [4, 5]. Collectively, these findings provide insight into leukemogenesis, fusion oncoproteins and cooperating mutations defining disease phenotypes.

Furthermore, our case demonstrates the recently described phenotype‐genotype association, i.e., the genotype of MPAL being associated with immunophenotypic lineage predominance [8], as seen in our case of B/T MPAL with T‐lineage predominance and cooperative gene alterations characteristic of T‐ALL. This association has clinical implications on the selection of appropriate induction therapy in MPAL, B‐ALL‐, T‐ALL‐, or AML‐based regimen [13]. Our patient was initially treated with T‐lineage‐matched induction therapy but experienced induction failure and achieved remission following consolidation chemotherapy (COG AALL0434 with nelarabine). This reflects the aggressiveness of *NUP98* rearranged leukemias and urges the need for novel therapy targeting NUP98 fusion. Molecular dependency of *KMT2A* has been demonstrated in *NUP98* fusion protein‐driven leukemogenesis [5]. Menin inhibitors have shown anti‐leukemia efficacy in *KMT2A*‐rearranged leukemia [15]. The efficacy of menin inhibitors remains to be explored in leukemia with *NUP98::BPTF* fusion protein.

From diagnostic perspective, our case illustrates a discrepancy between the WHO‐HEM5 and the International Consensus Classification (ICC). While this case met the diagnostic criteria of B/T‐T MPAL, it would be classified as T‐ALL with a comment on the minor population of B‐lymphoblasts per the ICC guideline that requires the smaller neoplastic blast population to be at least 5% of the total cells [1, 2]. Whether using the ICC or the WHO‐HEM5 criteria, it is essential to report a minor population of leukemic blasts from a divergent lineage, as this population may become predominant after therapy and have significant therapeutic and prognostic implications [16, 17]. Although optimal induction chemotherapy has not yet been well established in B/T MPAL, current guidelines recommend the ALL‐type induction regimen, most often guided by the dominant immunophenotype [3, 18]; our patient was effectively treated following this guideline as described above.

In summary, this is the first case study that characterizes the clinicopathologic and genomic features of B/T MPAL with *NUP98::BPTF* and provides insights into leukemogenesis. Future collaborative studies are necessary to better understand leukemia pathogenesis and to develop biologically guided therapies.

## Author Contributions

VN, LH, and WC conceptualised the case study and wrote the original draft. All authors critically revised the manuscript and approved the final version of the manuscript.

## Ethics Statement

All procedures performed in this case study were part of the clinical management in accordance with the ethical standards.

## Consent

Waived per approved protocol (IRB STU 122013–023).

## Conflicts of Interest

The authors declare no conflicts of interest.

## Data Availability

The data that support the findings are available from the corresponding author upon reasonable request.
